# Epigenetic regulation of transcription factors involved in NLRP3 inflammasome and NF-kB signaling pathways

**DOI:** 10.3389/fimmu.2025.1529756

**Published:** 2025-02-19

**Authors:** John Kaszycki, Minji Kim

**Affiliations:** ^1^ Department of Biological Sciences, University of Connecticut, Storrs, CT, United States; ^2^ School of Pharmacy, University of Connecticut, Storrs, CT, United States

**Keywords:** epigenetics, inflammation, NLRP3 inflammasome, NF-κB signaling, chromatin remodeling

## Abstract

The NLRP3 inflammasome and NF-κB signaling pathways play crucial roles in orchestrating inflammation and immune defense.​ This review explores the intricate relationship between these pathways and epigenetic regulation, a field of growing importance in understanding immune responses. Epigenetic modifications, including DNA methylation, histone modifications, and non-coding RNAs (ncRNAs), significantly influence the activity of genes involved in these pathways, thereby modulating inflammatory responses. The review provides a comprehensive overview of current research on how epigenetic mechanisms interact with and regulate the NLRP3 inflammasome and NF-κB signaling pathways. It delves into advanced epigenetic concepts such as RNA modifications and 3D genome organization, and their impact on immune regulation. Furthermore, the implications of these findings for developing novel therapeutic strategies targeting epigenetic regulators in inflammatory diseases are discussed. By synthesizing recent advancements in this rapidly evolving field, this review underscores the critical role of epigenetic regulation in immune signaling and highlights the potential for epigenetic-based therapies in treating a wide range of inflammatory conditions, including autoimmune disorders and cancer.

## 1 Introduction

The intricate mechanisms governing immune responses are fundamental to the survival and well-being of organisms. Among the many pathways that regulate these responses, the NLRP3 inflammasome and NF-κB signaling pathways are particularly critical due to their roles in orchestrating inflammation and immune defense (1). The NLRP3 inflammasome, a multi-protein complex, is essential in detecting pathogenic microorganisms and cellular stress, leading to the activation of inflammatory responses. Meanwhile, the NF-κB pathway is a central mediator of immune responses, inflammation, and cell survival (2). Both pathways are intricately connected, with cross-regulatory mechanisms ensuring the fine-tuning of immune responses. Dysregulation of these pathways is implicated in a wide range of inflammatory diseases, including autoimmune disorders, metabolic syndromes, and cancers (3, 4).

In recent years, there has been growing recognition that the NLRP3 inflammasome and NF-κB signaling pathways are regulated not only by genetic factors but also by epigenetic modifications—heritable changes in gene expression that do not involve alterations in the DNA sequence. Epigenetic regulation encompasses a range of mechanisms, including DNA methylation, histone modifications, and the involvement of ncRNAs, all of which contribute to the dynamic control of gene expression. These modifications can influence the accessibility of transcription factors to DNA, thereby modulating the transcriptional activity of genes involved in immune responses (5).

The NLRP3 inflammasome and NF-κB signaling pathways are particularly sensitive to epigenetic changes, which can either exacerbate or mitigate inflammatory responses. For example, aberrant DNA methylation patterns have been associated with chronic inflammation and autoimmune diseases, while histone modifications can either promote or repress the transcription of pro-inflammatory genes (6, 7). Furthermore, ncRNAs, including microRNAs (miRNAs), long non-coding RNAs (lncRNAs), and circular RNAs (circRNAs), have been shown to play crucial roles in the post-transcriptional regulation of genes within these pathways (8).

This review aims to provide a comprehensive overview of the current understanding of epigenetic regulation in the context of the NLRP3 inflammasome and NF-κB signaling pathways. We will explore the mechanisms by which epigenetic modifications influence these pathways, focusing on the interplay between epigenetics and transcription factors. The review will also examine the roles of advanced epigenetic mechanisms, such as RNA modifications and 3D genome organization, in regulating immune responses. Additionally, we will discuss the implications of these findings for the development of novel therapeutic strategies targeting epigenetic regulators, with a focus on inflammatory diseases such as rheumatoid arthritis (RA), inflammatory bowel disease, systemic lupus erythematosus, and cancer (9).

By synthesizing recent research in this rapidly evolving field, this review seeks to highlight the critical role of epigenetic regulation in modulating immune signaling pathways and to underscore the potential for epigenetic therapies in treating inflammatory diseases. Understanding the complex interactions between epigenetic modifications and immune signaling pathways will be crucial for developing more effective and targeted therapies for a wide range of inflammatory conditions.

## 2 Epigenetic mechanisms in immune regulation

Epigenetic mechanisms play a critical role in regulating immune responses by modulating the expression of genes involved in the development, differentiation, and function of immune cells. These mechanisms, which include DNA methylation, histone modifications, and the action of ncRNAs, provide a layer of control that allows immune cells to respond dynamically to environmental stimuli and maintain immune homeostasis (10). In recent years, research has uncovered the significant impact of these epigenetic processes on immune regulation, revealing their essential roles in both normal immune function and the pathogenesis of inflammatory and autoimmune diseases (11).

DNA methylation involves the addition of a methyl group to the 5’ position of cytosine residues within CpG dinucleotides, a process catalyzed by DNA methyltransferases (DNMTs), including DNMT1, DNMT3A, and DNMT3B (12). This modification typically results in transcriptional repression, as the methylation of CpG islands in gene promoter regions can inhibit the binding of transcription factors or recruit repressive complexes that promote chromatin condensation (13). DNA methylation is crucial for regulating the differentiation and function of immune cells, such as T cells, B cells, and macrophages (14).

During T cell development, DNA methylation patterns are dynamically regulated to control the expression of key transcription factors that drive the differentiation of naïve CD4+ T cells into specialized subsets, including Th1, Th2, Th17, and regulatory T cells (Tregs). For instance, the hypermethylation of the Ifng gene, which encodes interferon-gamma (IFN-γ), is associated with the suppression of Th1 differentiation, while hypomethylation of this gene in differentiated Th1 cells leads to its active expression. This process is crucial for maintaining the balance between pro-inflammatory and anti-inflammatory T cell subsets, which is necessary for a proper immune response (15).

Aberrant DNA methylation has been linked to the pathogenesis of several autoimmune diseases, where the improper regulation of immune cell differentiation and function contributes to chronic inflammation and tissue damage. For example, in patients with SLE, hypomethylation of the CD11a gene in CD4+ T cells leads to its overexpression, which is associated with increased autoreactivity and the production of autoantibodies (16). Similarly, in RA, hypomethylation of pro-inflammatory cytokine genes, such as TNF and IL6, has been observed, leading to their overexpression and the promotion of inflammatory responses (17).

Histone modifications represent another key epigenetic mechanism that influences immune regulation. Histones, which are the protein components of chromatin, can undergo various post-translational modifications, including acetylation, methylation, phosphorylation, and ubiquitination. These modifications occur primarily on the N-terminal tails of histones H3 and H4 and play a crucial role in determining chromatin structure and gene expression (18). Acetylation of histones, mediated by histone acetyltransferases (HATs), is generally associated with transcriptional activation. This process reduces the positive charge on histones, weakening their interaction with DNA and resulting in a more open chromatin structure that is accessible to transcription factors (19). Conversely, histone deacetylation, catalyzed by histone deacetylases (HDACs), is linked to transcriptional repression through the condensation of chromatin (18).

In immune cells, histone modifications are essential for the rapid and dynamic regulation of gene expression in response to external stimuli. For instance, upon activation of macrophages by bacterial components, such as lipopolysaccharide (LPS), there is a rapid increase in histone acetylation at the promoters of pro-inflammatory genes like TNF and IL6, facilitating their transcription and the subsequent inflammatory response (20). Histone methylation, another key modification, can either activate or repress transcription depending on the specific residues modified. For example, trimethylation of histone H3 lysine 4 (H3K4me3) is associated with active gene transcription and is enriched at the promoters of genes involved in T cell activation and differentiation (21). Conversely, trimethylation of histone H3 lysine 27 (H3K27me3) is a marker of transcriptional repression and is often found at the promoters of genes that need to be silenced to prevent inappropriate immune responses (7, 22).

The dysregulation of histone modifications has been implicated in the development of chronic inflammatory and autoimmune diseases. For instance, in SLE, global hypoacetylation of histones has been observed, which is associated with the repression of key regulatory genes and the promotion of autoimmunity (23). Targeting histone-modifying enzymes, such as HDAC inhibitors, has emerged as a potential therapeutic strategy for modulating immune responses and treating inflammatory diseases (6). HDAC inhibitors can restore the balance of histone acetylation and have shown promise in preclinical models of autoimmune diseases, including RA and multiple sclerosis (MS) (24).

NcRNAs have also emerged as critical regulators of immune responses. NcRNAs include miRNAs, lncRNAs, and circRNAs, all of which play roles in regulating gene expression at the transcriptional and post-transcriptional levels. MiRNAs are small, approximately 22-nucleotide long RNA molecules that regulate gene expression by binding to complementary sequences in the 3’ untranslated regions (UTRs) of target mRNAs, leading to mRNA degradation or translational repression. In immune cells, miRNAs are involved in regulating processes such as T cell differentiation, cytokine production, and the resolution of inflammation (25). For example, miR-146a is a well-known miRNA that acts as a negative regulator of the NF-κB signaling pathway, modulating the inflammatory response by targeting key components of the pathway, such as TRAF6 and IRAK1 (26).

LncRNAs are a diverse group of ncRNAs longer than 200 nucleotides, involved in various aspects of gene regulation, including chromatin remodeling, transcriptional control, and post-transcriptional processing. In the immune system, lncRNAs have been shown to regulate the expression of key immune genes and modulate the activation and differentiation of immune cells. For instance, the lncRNA NEAT1 has been implicated in the regulation of the inflammatory response by controlling the assembly of paraspeckles, nuclear bodies involved in the retention of mRNAs encoding pro-inflammatory cytokines (27). Dysregulation of lncRNAs has been linked to inflammatory diseases, where altered expression of these RNAs can contribute to the pathogenesis of conditions such as SLE and inflammatory bowel disease (IBD) (28).

CircRNAs, which form covalently closed loop structures, add another layer of complexity to the regulation of gene expression in immune cells. These molecules can function as miRNA sponges, sequestering miRNAs and preventing them from binding to their target mRNAs, thereby modulating gene expression. For instance, circ_0005075 has been shown to regulate the expression of IL-6 and IL-1β in macrophages by sponging miR-431-5p, thereby modulating the inflammatory response (29). The dysregulation of circRNAs has been associated with various inflammatory conditions, and their unique structure makes them attractive targets for therapeutic intervention.

## 3 Interplay between epigenetics and transcription factors in NLRP3 and NF-κB pathways

The interplay between epigenetic mechanisms and transcription factors is central to the regulation of the NLRP3 inflammasome and NF-κB signaling pathways, both of which are critical in orchestrating immune responses and inflammation. Epigenetic modifications such as DNA methylation, histone modifications, and ncRNAs influence the activity of transcription factors, which in turn modulate the expression of genes involved in these pathways. This dynamic interaction ensures that immune responses are tightly regulated and can be adapted to various stimuli, thereby maintaining immune homeostasis and preventing excessive inflammation (5).

DNA methylation plays a crucial role in modulating the binding of transcription factors to the promoters of genes involved in the NLRP3 and NF-κB pathways. For instance, the methylation status of CpG sites within gene promoters can either facilitate or inhibit the binding of transcription factors, thereby regulating gene expression. In the context of the NLRP3 inflammasome, the transcription factor NF-κB is a key regulator that controls the expression of NLRP3 itself, as well as pro-inflammatory cytokines such as IL-1β and IL-18 (30). Aberrant DNA methylation patterns within the promoters of these genes can lead to either the suppression or overexpression of NLRP3 and its associated cytokines, contributing to chronic inflammation and autoimmune diseases. For example, hypomethylation of the NLRP3 promoter has been observed in patients with inflammatory bowel disease (IBD), leading to enhanced expression of NLRP3 and increased inflammation (31).

Histone modifications are another critical epigenetic mechanism that influences the activity of transcription factors in the NLRP3 and NF-κB pathways. Acetylation of histones, particularly histone H3 and H4, is associated with an open chromatin structure that allows transcription factors to access gene promoters and initiate transcription (32). The acetylation of histones at the promoters of NF-κB target genes, such as those encoding pro-inflammatory cytokines and chemokines, enhances the recruitment of NF-κB to these sites and promotes the transcription of inflammatory genes. Conversely, deacetylation of histones by HDACs can lead to chromatin condensation and transcriptional repression, thereby limiting the activity of NF-κB and reducing inflammation (33).

Methylation of histones also plays a pivotal role in regulating the expression of genes involved in the NLRP3 and NF-κB pathways. For example, trimethylation of histone H3 at lysine 4 (H3K4me3) is a marker of active transcription and is often found at the promoters of genes involved in inflammatory responses. This modification facilitates the binding of NF-κB to its target genes, thereby promoting the expression of NLRP3 and pro-inflammatory cytokines (34). On the other hand, trimethylation of histone H3 at lysine 27 (H3K27me3) is a repressive mark that is associated with the silencing of inflammatory genes (35). The balance between these activating and repressive histone marks is crucial for the proper regulation of the NLRP3 inflammasome and NF-κB signaling, and dysregulation of this balance can lead to chronic inflammation and disease (36).

NcRNAs, particularly miRNAs and lncRNAs, also play a significant role in modulating the activity of transcription factors in the NLRP3 and NF-κB pathways. MiRNAs are short, ncRNAs that regulate gene expression post-transcriptionally by binding to the 3’ untranslated regions (UTRs) of target mRNAs, leading to their degradation or translational repression. In the NF-κB pathway, miRNAs such as miR-146a and miR-155 have been shown to regulate the expression of key components of the pathway, including TRAF6 and IRAK1, which are upstream activators of NF-κB (37). By modulating the levels of these components, miRNAs can either enhance or suppress NF-κB activity, thereby influencing the downstream expression of inflammatory genes (38).

LncRNAs, on the other hand, can modulate the activity of transcription factors through a variety of mechanisms, including acting as molecular scaffolds, decoys, or guides for chromatin-modifying complexes. In the context of the NF-κB pathway, lncRNAs such as NKILA have been shown to interact directly with NF-κB, inhibiting its activity by preventing its phosphorylation and subsequent nuclear translocation. This interaction highlights the complexity of the regulatory networks involving lncRNAs and transcription factors, and underscores the importance of these non-coding RNAs in controlling inflammatory responses (39).

## 4 Role of long non-coding RNAs and circular RNAs

Long non-coding RNAs (lncRNAs) and circRNAs have emerged as significant regulators in various biological processes, including immune responses and inflammation. Unlike protein-coding RNAs, these ncRNAs do not encode proteins but instead function through diverse mechanisms to modulate gene expression. Their roles in the regulation of the NLRP3 inflammasome and NF-κB signaling pathways, which are central to inflammation and immune responses, have garnered increasing attention in recent years. Both lncRNAs and circRNAs have been shown to interact with key components of these pathways, influencing the activation and suppression of inflammatory genes and thus contributing to the fine-tuning of immune responses (40).

LncRNAs are a heterogeneous class of ncRNAs that are longer than 200 nucleotides. They can regulate gene expression at various levels, including chromatin remodeling, transcriptional control, and post-transcriptional processing. In the context of the NF-κB pathway, lncRNAs have been found to interact directly with NF-κB or its regulators, thereby modulating the pathway’s activity. For instance, the lncRNA NKILA (NF-κB interacting lncRNA) has been shown to inhibit NF-κB signaling by binding to the NF-κB/IκB complex, preventing the phosphorylation of IκB and the subsequent nuclear translocation of NF-κB. This inhibition reduces the expression of NF-κB target genes, which are typically involved in promoting inflammation. The role of NKILA illustrates how lncRNAs can act as negative regulators of inflammation, potentially providing a protective mechanism against chronic inflammatory conditions (41).

Another example of a lncRNA involved in the regulation of the NF-κB pathway is MALAT1 (Metastasis Associated Lung Adenocarcinoma Transcript 1). MALAT1 has been shown to enhance NF-κB signaling by promoting the nuclear translocation of the NF-κB subunit p65, thereby increasing the transcription of pro-inflammatory genes. This lncRNA serves as a positive regulator of inflammation and has been implicated in various inflammatory diseases, including RA and atherosclerosis. The contrasting roles of lncRNAs like NKILA and MALAT1 highlight the complexity of lncRNA-mediated regulation of the NF-κB pathway, where different lncRNAs can either suppress or promote inflammation depending on the context (2).

**Figure 1:**
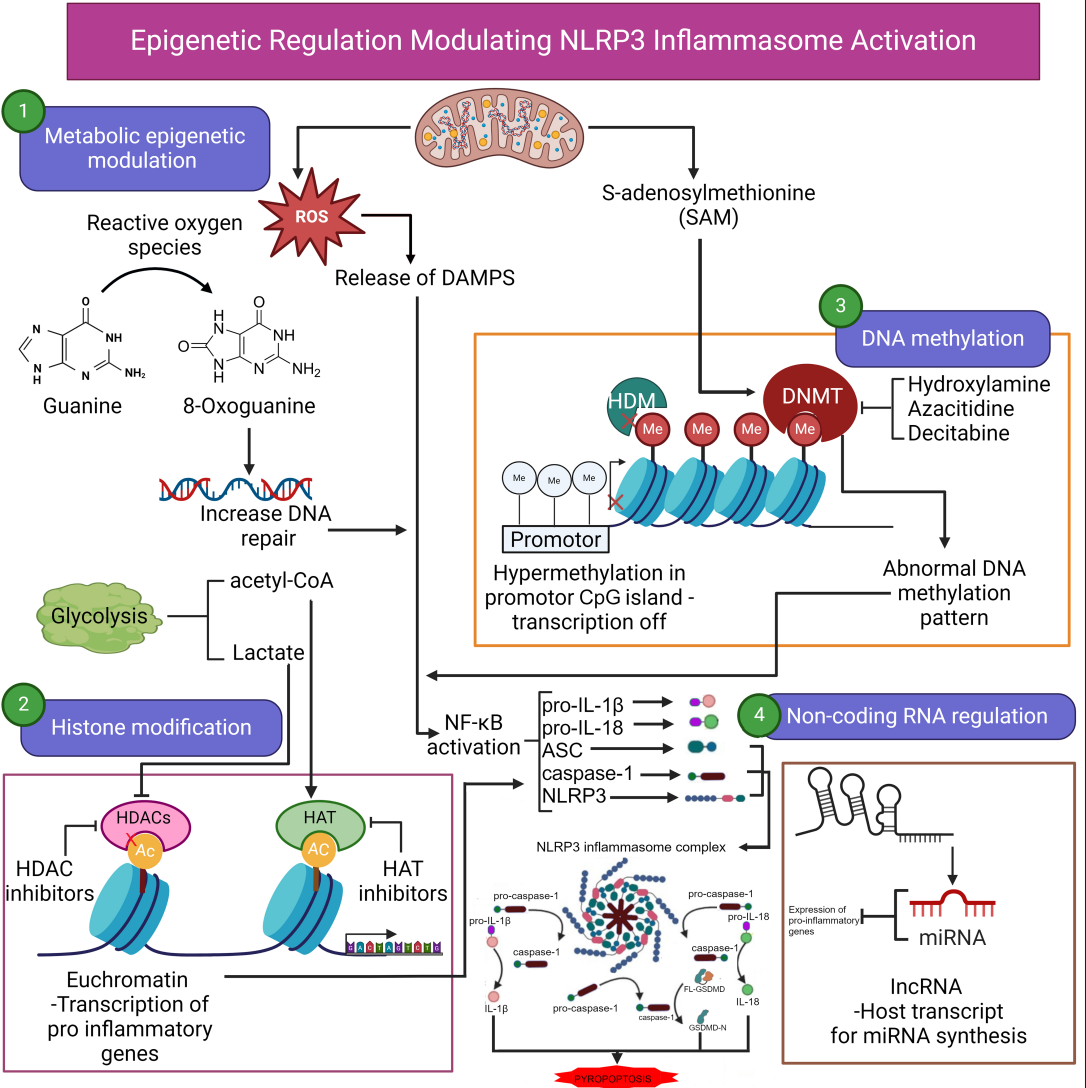
Epigenetic regulation modulating NLRP3 inflammasome activation. Epigenetic regulation of NLRP3 inflammasome activation involves several key processes, including metabolic epigenetic modulation, histone modification, DNA methylation, and noncoding RNA regulation. Reactive oxygen species (ROS) generated during metabolic activities lead to oxidative DNA damage (8-oxoguanine), prompting increased DNA repair. Lactate production through glycolysis also contributes to acetylation of histones, facilitating transcriptional activation of pro-inflammatory genes. ROS can also induce the release of damage-associated molecular patterns (DAMPs), further enhancing inflammation. Histone modifications play a crucial role in the transcription of inflammasome-associated genes. Histone acetyltransferases (HATs) promote the acetylation of histones, resulting in an open chromatin configuration (euchromatin), which permits transcription of pro-inflammatory genes. In contrast, histone deacetylases (HDACs) compact chromatin, suppressing transcription. DNA methylation by DNA methyltransferases (DNMTs) results in hypermethylation of promoter regions (e.g., CpG islands), repressing transcription. Abnormal methylation patterns are associated with aberrant inflammasome activation. Agents such as hydroxylamine, azacitidine, and decitabine can inhibit DNMTs, potentially reversing hypermethylation and restoring normal gene expression. Noncoding RNAs, including microRNAs (miRNAs) and long noncoding RNAs (lncRNAs), also regulate the expression of NLRP3 inflammasome genes. miRNAs can target mRNA for degradation or inhibit translation. These epigenetic factors converge to modulate NLRP3 inflammasome activation, contributing to the inflammatory response. Ac, acetylated; ASC, apoptosis-associated speck-like protein containing a CARD; CpG, cytosine-phosphate-guanine; DNMT, DNA methyltransferase; DAMPs, damage-associated molecular patterns; HAT, histone acetyltransferase; HDAC, histone deacetylase; HDM, histone demethylase; IL, interleukin; lncRNA, long noncoding RNA; Me, methylated; miRNA, microRNA; NF-κB, nuclear transcription factor kappa β; NLRP3, NOD-like receptor family pyrin domain containing 3 inflammasome; ROS, reactive oxygen species. Continued arrows indicate “stimulation”, and blocked arrows indicate “inhibition”. Created in BioRender.com.

LncRNAs also play a crucial role in the regulation of the NLRP3 inflammasome, a multi-protein complex that is activated in response to cellular stress and infection, leading to the production of pro-inflammatory cytokines such as IL-1β and IL-18 (**Figure 1**). The lncRNA MIAT (Myocardial Infarction Associated Transcript) has been shown to regulate the NLRP3 inflammasome in macrophages. MIAT promotes the activation of the NLRP3 inflammasome by sponging miR-214-3p, a microRNA that negatively regulates NLRP3 expression (42). By sequestering miR-214-3p, MIAT prevents the repression of NLRP3, thereby facilitating inflammasome activation and the subsequent inflammatory response. This interaction exemplifies the role of lncRNAs as molecular sponges, a mechanism through which they can modulate the activity of other regulatory RNAs and influence inflammation (43).

CircRNAs, another class of ncRNAs, are formed through a process called back-splicing, where the 3’ and 5’ ends of an RNA molecule are covalently linked to form a closed loop structure. This unique structure makes circRNAs resistant to exonuclease-mediated degradation, allowing them to persist in cells and potentially exert long-lasting regulatory effects. CircRNAs can function as microRNA (miRNA) sponges, sequestering miRNAs and preventing them from binding to their target mRNAs, thereby modulating gene expression. In the NF-κB pathway, circRNAs such as circ_0005075 have been identified as regulators of inflammation. Circ_0005075 acts as a sponge for miR-431-5p, a miRNA that negatively regulates the expression of IL-6 and IL-1β, two key pro-inflammatory cytokines (44). By sponging miR-431-5p, circ_0005075 promotes the expression of these cytokines, thereby enhancing the inflammatory response. This example highlights the role of circRNAs in fine-tuning the expression of inflammatory genes through miRNA sequestration (45).

CircRNAs have also been implicated in the regulation of the NLRP3 inflammasome. For example, circ_0003204 has been shown to modulate the activation of the NLRP3 inflammasome in macrophages by sponging miR-138-5p, which targets NLRP3 for degradation (46). By sequestering miR-138-5p, circ_0003204 prevents the degradation of NLRP3, thereby promoting inflammasome activation and the production of IL-1β. This mechanism underscores the importance of circRNAs in regulating the balance between immune activation and suppression, with implications for the development and resolution of inflammation (47).

The dysregulation of lncRNAs and circRNAs has been associated with various inflammatory and autoimmune diseases, highlighting their potential as biomarkers and therapeutic targets. For instance, elevated levels of MALAT1 and circ_0005075 have been observed in patients with RA, suggesting that these ncRNAs could serve as indicators of disease activity and potential targets for intervention (48). Similarly, the overexpression of MIAT has been linked to increased NLRP3 inflammasome activity in cardiovascular diseases, making it a potential target for therapeutic strategies aimed at reducing inflammation (49).

## 5 Epigenetic regulation through 3D genome organization

Epigenetic regulation through 3D genome organization is an emerging field that highlights the importance of the spatial arrangement of chromatin within the nucleus in controlling gene expression. This spatial organization plays a crucial role in the regulation of genes involved in immune responses, including those associated with the NLRP3 inflammasome and NF-κB signaling pathways. The dynamic nature of 3D genome organization allows for the precise and context-specific regulation of gene expression, which is essential for maintaining immune homeostasis and preventing aberrant inflammatory responses (50).

One of the fundamental units of 3D genome organization is the topologically associating domain (TAD). TADs are contiguous regions of the genome that interact more frequently with themselves than with neighboring regions, creating a modular organization of the genome (51). The boundaries of TADs are often defined by the binding of architectural proteins such as CTCF and cohesin, which play a key role in maintaining the structural integrity of these domains (52). This spatial proximity is particularly important in the regulation of genes involved in immune responses, where rapid and precise control of gene expression is required (53).

In the context of the NF-κB signaling pathway, 3D genome organization has been shown to play a critical role in the regulation of inflammatory gene expression. Upon activation of NF-κB, there is a reorganization of chromatin architecture that brings enhancers into close contact with the promoters of NF-κB target genes, thereby facilitating their transcription (54). This reorganization is mediated by the recruitment of NF-κB to specific genomic loci, where it interacts with other transcription factors and coactivators to promote chromatin looping. The formation of these loops allows for the coordinated regulation of multiple genes involved in the inflammatory response, ensuring a rapid and robust immune reaction (55).

Similarly, the activation of the NLRP3 inflammasome is also influenced by 3D genome organization. Recent studies have shown that the genes encoding components of the NLRP3 inflammasome, such as NLRP3 and IL1B, are located within specific TADs that facilitate their coordinated regulation (56). The spatial organization of these genes within TADs allows for the efficient activation of the inflammasome in response to stimuli, as the necessary transcriptional machinery and regulatory elements are brought into close proximity. This organization ensures that the expression of inflammasome components is tightly controlled, preventing excessive inflammation and tissue damage (57).

The impact of 3D genome organization on gene regulation is further exemplified by the role of chromatin loops in controlling the expression of cytokines and chemokines. For instance, the TNF locus, which encodes the pro-inflammatory cytokine TNF-α, is located within a TAD that undergoes significant remodeling upon activation of NF-κB (57). This remodeling involves the formation of new chromatin loops that bring enhancers into contact with the TNF promoter, thereby enhancing its transcription. The dynamic nature of these loops allows for the rapid induction of TNF-α expression in response to infection or injury, highlighting the importance of 3D genome organization in the regulation of inflammatory responses (57).

Epigenetic modifications such as histone modifications and DNA methylation also play a role in regulating 3D genome organization. For example, the methylation of histone H3 at lysine 9 (H3K9me3) is associated with the formation of repressive chromatin structures that limit the accessibility of transcriptional machinery to specific genomic regions (58). These repressive structures can influence the formation of TADs and chromatin loops, thereby regulating the expression of genes involved in immune responses. The interplay between epigenetic modifications and 3D genome organization adds an additional layer of complexity to the regulation of gene expression, ensuring that immune responses are tightly controlled (59).

Moreover, recent advances in techniques such as Hi-C and chromatin interaction analysis by paired-end tag sequencing (ChIA-PET) have provided insights into the role of 3D genome organization in gene regulation (60). These techniques have revealed that the spatial organization of the genome is highly dynamic, with chromatin loops and TADs undergoing constant remodeling in response to external stimuli. This dynamic nature of 3D genome organization allows for the precise regulation of immune genes, enabling cells to rapidly respond to changes in their environment (60).

The dysregulation of 3D genome organization has been implicated in various inflammatory and autoimmune diseases. For example, alterations in the spatial organization of the TNF locus have been associated with chronic inflammatory conditions such as RA and Crohn’s disease (61). These alterations can lead to the inappropriate activation of inflammatory genes, contributing to the pathogenesis of these diseases. Understanding the role of 3D genome organization in the regulation of immune responses offers potential therapeutic targets for modulating inflammation and treating autoimmune diseases (62).

## 6 Metabolic reprogramming and epigenetics in inflammation

Metabolic reprogramming and epigenetics are closely intertwined processes that play a significant role in the regulation of inflammation. Immune cells undergo extensive metabolic changes in response to activation, which in turn influence their function, differentiation, and the nature of the inflammatory response. These metabolic shifts are not merely a consequence of cellular activation; they actively contribute to the regulation of gene expression through epigenetic mechanisms. The interplay between metabolism and epigenetics provides a critical layer of control over immune responses, ensuring that inflammation is appropriately regulated to protect the host without causing excessive tissue damage (63).

One of the key aspects of metabolic reprogramming in immune cells is the switch from oxidative phosphorylation to glycolysis upon activation. This shift, often referred to as the Warburg effect, is characterized by an increased reliance on glycolysis even in the presence of sufficient oxygen (64). Glycolysis provides rapid ATP production and generates intermediates that are essential for biosynthetic processes, supporting the high energy and biosynthetic demands of activated immune cells. However, this metabolic reprogramming also has profound effects on epigenetic regulation. For instance, the increased flux through glycolysis leads to the accumulation of metabolites such as acetyl-CoA and lactate, which can influence histone modifications and gene expression (65).

Acetyl-CoA, a key intermediate in glycolysis, serves as a substrate for histone acetyltransferases (HATs) that acetylate histones and promote gene transcription (66). The availability of acetyl-CoA directly affects the levels of histone acetylation, linking cellular metabolism to the regulation of gene expression. In activated immune cells, the increased production of acetyl-CoA enhances histone acetylation at the promoters of pro-inflammatory genes, facilitating their transcription. For example, in macrophages, histone acetylation is increased at the promoters of genes encoding cytokines such as IL-6 and TNF-α, driving the expression of these key inflammatory mediators (67). This acetylation-dependent regulation of gene expression underscores the role of metabolic reprogramming in shaping the inflammatory response through epigenetic mechanisms (68).

Another metabolite that plays a crucial role in the link between metabolism and epigenetics is lactate. During glycolysis, pyruvate is converted to lactate, leading to the accumulation of lactate in the cell. Lactate can inhibit histone deacetylases (HDACs), enzymes that remove acetyl groups from histones and suppress gene expression (69). By inhibiting HDACs, lactate promotes the maintenance of histone acetylation, thereby sustaining the transcription of pro-inflammatory genes. This mechanism highlights how metabolic byproducts can modulate the epigenetic landscape, influencing the persistence and intensity of inflammatory responses (70).

In addition to glycolysis, other metabolic pathways such as the tricarboxylic acid (TCA) cycle and fatty acid oxidation also contribute to the epigenetic regulation of inflammation. The TCA cycle produces α-ketoglutarate, a cofactor for DNA and histone demethylases that remove methyl groups from DNA and histones, respectively. This demethylation activity is crucial for the activation of anti-inflammatory genes and the resolution of inflammation. For example, in regulatory T cells (Tregs), α-ketoglutarate promotes the demethylation of the *Foxp3* gene, which is essential for the suppressive function of Tregs and the control of inflammation (71). The role of the TCA cycle in generating metabolites that influence epigenetic modifications underscores the importance of metabolic pathways in regulating immune responses at the epigenetic level (72).

Fatty acid oxidation is another metabolic pathway that influences epigenetic regulation in immune cells. In contrast to glycolysis, which is associated with pro-inflammatory responses, fatty acid oxidation is linked to the generation of anti-inflammatory immune cell phenotypes. For instance, in macrophages, the switch from glycolysis to fatty acid oxidation is associated with the activation of anti-inflammatory programs and the resolution of inflammation (73). This metabolic shift is accompanied by changes in histone modifications, such as increased histone methylation at anti-inflammatory gene loci, which suppresses the expression of pro-inflammatory genes. The interplay between fatty acid metabolism and histone modifications highlights the role of metabolic reprogramming in controlling the balance between pro- and anti-inflammatory responses (74).

The link between metabolism and epigenetics is not limited to histone modifications but also extends to DNA methylation. S-adenosylmethionine (SAM), a key methyl donor for DNA methylation, is generated from the amino acid methionine in a process that is closely tied to cellular metabolism. In conditions of metabolic stress, such as nutrient deprivation or hypoxia, the production of SAM can be altered, leading to changes in DNA methylation patterns that impact immune cell function and the inflammatory response (75). This connection between metabolism and DNA methylation further illustrates the intricate relationship between cellular metabolism and epigenetic regulation in controlling inflammation (76).

## 7 Inter-organellar communication in epigenetic regulation

Inter-organellar communication plays a crucial role in the regulation of cellular functions, including the epigenetic control of gene expression. The interaction between organelles such as the mitochondria, endoplasmic reticulum (ER), and nucleus is essential for coordinating cellular responses to various stimuli, including those that influence inflammation and immune responses. Recent research has highlighted how the crosstalk between these organelles contributes to the regulation of epigenetic modifications, thereby influencing the activity of key signaling pathways such as the NLRP3 inflammasome and NF-κB (77).

Mitochondria, often referred to as the powerhouse of the cell, are central to energy production and metabolic regulation. Beyond their metabolic functions, mitochondria also play a critical role in the regulation of epigenetic mechanisms through the generation of metabolites and reactive oxygen species (ROS). Mitochondrial metabolites, such as acetyl-CoA, α-ketoglutarate, and S-adenosylmethionine (SAM), are essential substrates for enzymes that catalyze epigenetic modifications, including histone acetylation and DNA methylation (78). The production of these metabolites within the mitochondria is closely linked to the cell’s metabolic state, which in turn influences the epigenetic landscape. For example, the mitochondrial production of acetyl-CoA is directly tied to histone acetylation levels, impacting the expression of genes involved in inflammation and immune responses (79).

Reactive oxygen species (ROS), another byproduct of mitochondrial metabolism, also play a significant role in epigenetic regulation. ROS can induce oxidative modifications of DNA and histones, leading to changes in gene expression (80). For instance, the oxidation of guanine to 8-oxoguanine in DNA can lead to the recruitment of the base excision repair machinery, which in turn can influence chromatin structure and gene transcription (81). Moreover, ROS-mediated modifications of histones, such as the oxidation of histone H3 at methionine 56, can alter the binding affinity of chromatin to DNA, affecting the accessibility of transcription factors and thus gene expression. This oxidative regulation of epigenetic modifications underscores the critical role of mitochondria in linking cellular metabolism to gene regulation, particularly in the context of inflammation (82).

The endoplasmic reticulum (ER) is another organelle that plays a pivotal role in epigenetic regulation through its involvement in protein folding, calcium homeostasis, and lipid metabolism. The ER interacts closely with mitochondria through structures known as mitochondria-associated membranes (MAMs), which facilitate the transfer of calcium and lipids between these organelles (83). Calcium signaling between the ER and mitochondria is particularly important for the regulation of mitochondrial function and, consequently, the generation of metabolites that influence epigenetic modifications. For example, calcium flux between the ER and mitochondria can regulate the activity of mitochondrial enzymes involved in the TCA cycle, thereby influencing the production of α-ketoglutarate and other metabolites that impact DNA and histone demethylation (84).

In addition to calcium signaling, the ER also plays a role in the regulation of lipid metabolism, which can influence chromatin structure and gene expression. Lipid-derived molecules, such as phosphatidylinositol and its phosphorylated derivatives, can act as signaling molecules that influence the recruitment of chromatin modifiers to specific genomic loci, thereby regulating gene transcription (85). For instance, inositol phosphates produced by the ER can regulate the activity of chromatin remodelers such as SWI/SNF, which in turn modulates the expression of genes involved in immune responses (86).

Moreover, the ER stress response, which is activated in conditions of cellular stress, can also influence epigenetic regulation. ER stress leads to the activation of the unfolded protein response (UPR), a signaling pathway that helps restore ER homeostasis by modulating protein folding and degradation (87). The UPR can also influence gene expression by regulating the activity of transcription factors such as ATF6, XBP1, and CHOP, which in turn can affect the expression of genes involved in inflammation (88). Through its influence on transcription factors, the UPR links ER stress to epigenetic modifications, thereby contributing to the regulation of inflammatory responses (89).

Inter-organellar communication also extends to the nucleus, where signals from the mitochondria and ER are integrated to regulate gene expression. For example, mitochondrial dysfunction can lead to changes in the expression of nuclear-encoded genes involved in oxidative stress responses, such as those regulated by the transcription factor NRF2 (90). NRF2 activation leads to the transcription of antioxidant genes that help mitigate the effects of ROS, thereby protecting the cell from oxidative damage. The regulation of NRF2 by mitochondrial signals illustrates how inter-organellar communication can influence nuclear gene expression and, consequently, the cellular response to inflammation (91).

Similarly, the transfer of lipid signals from the ER to the nucleus can influence the activity of nuclear receptors such as peroxisome proliferator-activated receptors (PPARs), which regulate the expression of genes involved in lipid metabolism and inflammation (92). PPARs can recruit chromatin remodelers and histone modifiers to target gene promoters, thereby influencing the epigenetic landscape and modulating the inflammatory response. The involvement of PPARs in the regulation of inflammation through epigenetic mechanisms highlights the integrative role of inter-organellar communication in controlling immune responses (93).

## 8 Disease focus: epigenetic regulation in inflammatory diseases

Inflammation has a profound impact on the development and progression of many diseases (94–97). Epigenetic regulation plays a pivotal role in the pathogenesis of various inflammatory diseases by modulating the expression of genes involved in immune responses and inflammation. This regulation includes DNA methylation, histone modifications, and ncRNAs, all of which contribute to the complex interactions that determine the onset, progression, and severity of inflammatory conditions. Understanding the epigenetic mechanisms underlying these diseases provides insight into potential therapeutic targets and strategies for managing chronic inflammation and autoimmunity (98).

Rheumatoid arthritis (RA) is a chronic inflammatory disease characterized by persistent synovial inflammation and joint destruction. Epigenetic alterations, particularly DNA methylation and histone modifications, have been extensively studied in RA. Aberrant DNA methylation patterns in RA synovial fibroblasts (RASFs) contribute to the aggressive behavior of these cells, which are responsible for cartilage and bone destruction (99). Hypomethylation of pro-inflammatory cytokine genes, such as TNF and IL6, leads to their overexpression, exacerbating the inflammatory response in the joints (100). Additionally, histone modifications, such as increased histone acetylation at the promoters of these cytokines, further enhance their expression, perpetuating the chronic inflammation observed in RA (101). These epigenetic changes highlight the role of dysregulated gene expression in the pathogenesis of RA and suggest that targeting epigenetic modifiers could be a viable therapeutic strategy (17).

Inflammatory bowel disease (IBD), which includes Crohn’s disease (CD) and ulcerative colitis (UC), is another condition where epigenetic regulation plays a significant role. The chronic inflammation observed in IBD is driven by a complex interplay between genetic, environmental, and epigenetic factors. DNA methylation patterns in the intestinal mucosa of IBD patients differ significantly from those in healthy individuals, particularly in genes involved in immune regulation and barrier function (102). For example, hypomethylation of the TNFSF15 gene, which encodes a cytokine involved in T-cell activation, has been associated with increased expression and contributes to the pathogenesis of IBD (103). In addition to DNA methylation, histone modifications also play a crucial role in IBD. Aberrant histone acetylation patterns have been observed in the intestinal epithelium, leading to the dysregulation of genes involved in the immune response and epithelial barrier integrity (104). These findings underscore the importance of epigenetic regulation in maintaining intestinal homeostasis and suggest that epigenetic therapies could offer new avenues for IBD treatment (105).

Systemic lupus erythematosus (SLE) is a systemic autoimmune disease characterized by the production of autoantibodies and widespread inflammation affecting multiple organs. Epigenetic alterations, particularly DNA hypomethylation, have been implicated in the pathogenesis of SLE. Hypomethylation of DNA in CD4+ T cells from SLE patients leads to the overexpression of genes involved in autoimmunity, including ITGAL (CD11a) and CD70, which contribute to the activation of autoreactive T cells and the production of autoantibodies (16). Moreover, histone modifications, such as increased acetylation of histones H3 and H4, have been associated with the overexpression of pro-inflammatory cytokines and chemokines in SLE, contributing to the persistent inflammation observed in this disease (106). These epigenetic changes suggest that therapeutic strategies aimed at reversing DNA hypomethylation or modulating histone acetylation could be effective in managing SLE (107).

Cancer-related inflammation is another area where epigenetic regulation plays a crucial role. Chronic inflammation is a known risk factor for the development of certain cancers, and epigenetic alterations can exacerbate this process by promoting the expression of oncogenes and silencing tumor suppressor genes. For instance, in colorectal cancer, chronic inflammation is associated with the hypermethylation of the MLH1 gene, a key DNA mismatch repair gene, leading to its silencing and contributing to tumorigenesis (108). The interplay between epigenetic regulation and inflammation in cancer highlights the potential for epigenetic therapies to modulate inflammatory pathways and reduce cancer risk (109).

## 9 Epigenetic therapeutic strategies and future directions

**Figure 2:**
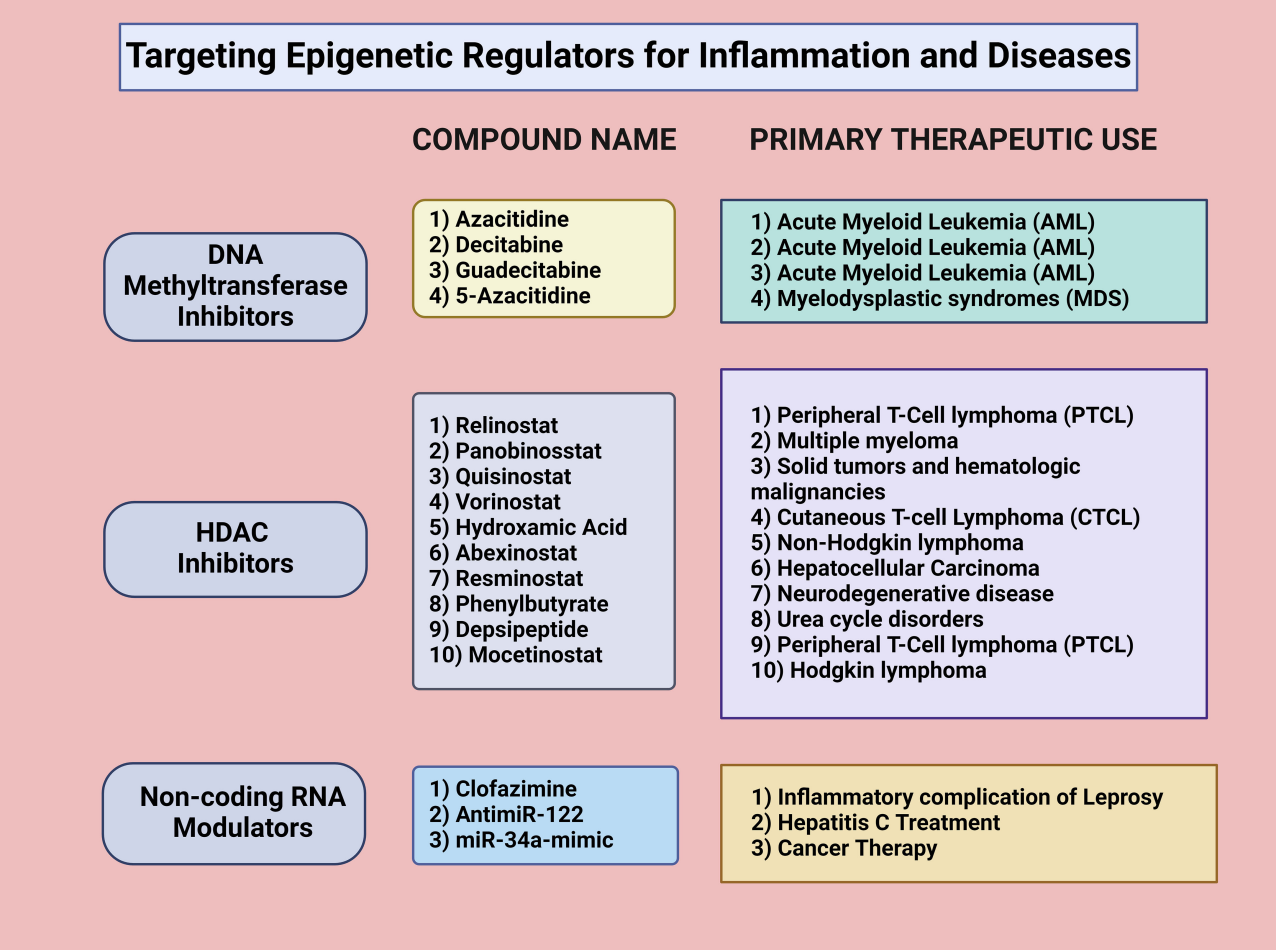
Targeting epigenetic regulators for inflammation and diseases. Targeting epigenetic regulators for inflammation and disease treatment involves various approaches. DNA methyltransferase inhibitors, such as azacitidine, decitabine, guadecitabine, and 5-azacitidine, are commonly used for acute myeloid leukemia (AML) and myelodysplastic syndromes (MDS). HDAC inhibitors, including Relinostat, Panobinostat, Quisinostat, and Yorinostat, modulate histone acetylation and deacetylation, affecting gene transcription in diseases like peripheral T-cell lymphoma (PTCL), multiple myeloma, and solid tumors. Noncoding RNA modulators, such as clofazimine, antimiR-122, and miR-34a-mimic, regulate gene expression post-transcriptionally and are applied in treating inflammatory conditions, hepatitis C, and cancer. Created in BioRender.com.

Epigenetic therapeutic strategies have emerged as promising approaches for the treatment of inflammatory diseases, given the central role of epigenetic regulation in controlling gene expression related to inflammation and immune responses. These strategies aim to modify epigenetic marks, such as DNA methylation, histone modifications, and ncRNAs, to restore normal gene expression patterns and ameliorate disease symptoms (**Figure 2**). Advances in epigenetic therapies, including the development of small molecule inhibitors, gene editing technologies, and personalized medicine approaches, offer new avenues for the treatment of chronic inflammatory conditions (5).

One of the most well-established epigenetic therapeutic strategies is the use of DNA methyltransferase inhibitors (DNMTis) to reverse aberrant DNA methylation patterns associated with inflammatory diseases. DNMTis, such as azacitidine and decitabine, inhibit the activity of DNA methyltransferases, leading to the demethylation of hypermethylated genes and the reactivation of their expression. These inhibitors are currently being evaluated in clinical trials for their efficacy in treating inflammatory conditions, showcasing their potential in reversing epigenetic alterations that contribute to immune dysregulation. Such trials aim to determine their safety, dosage, and therapeutic impact, paving the way for broader clinical applications (110, 111). These drugs have shown promise in the treatment of diseases where DNA hypermethylation contributes to pathogenesis, such as SLE (112). DNMTis have been explored as potential treatments to reverse these epigenetic changes, thereby reducing autoimmunity and inflammation (113).

Histone deacetylase inhibitors (HDACis) represent another class of epigenetic therapies with potential applications in treating inflammatory diseases. HDACis, such as vorinostat and trichostatin A, inhibit HDACs, leading to increased acetylation of histones and the activation of gene expression (114). By promoting the expression of anti-inflammatory genes and repressing the expression of pro-inflammatory genes, HDACis can help to restore the balance between pro- and anti-inflammatory responses. A phase I/II trial has been conducted to study the side effects and optimal dosage of vorinostat in preventing graft versus host disease in younger populations, including children, adolescents, and young adults (115, 116). Another trial involving vorinostat combined with standard treatments for glioblastoma concluded that although the primary efficacy objective was not met, vorinostat sensitivity and resistance signatures could help in patient selection (117). The ability of HDACis to modulate the epigenetic landscape and influence the expression of key inflammatory mediators makes them attractive candidates for the treatment of chronic inflammatory diseases (118).

The development of gene editing technologies, particularly CRISPR-Cas9, has opened new possibilities for targeted epigenetic therapies. CRISPR-Cas9 can be engineered to modify specific epigenetic marks at particular genomic loci, offering a highly precise approach to reprogramming gene expression (119). For example, CRISPR-based epigenome editing can be used to demethylate promoter regions of genes involved in immune regulation, thereby restoring their expression and ameliorating disease symptoms. This approach has the potential to correct epigenetic defects in a targeted manner, providing long-lasting therapeutic effects with minimal off-target effects. The use of CRISPR-Cas9 for epigenetic editing is still in its early stages, but it holds significant promise for the development of personalized therapies for inflammatory diseases (120).

NcRNAs, particularly miRNAs and lncRNAs, are also being explored as targets for epigenetic therapies. miRNAs can be modulated using antisense oligonucleotides (ASOs) or miRNA mimics to either inhibit or enhance their function (121). For example, in RA, miR-155, a pro-inflammatory miRNA, is upregulated and contributes to the pathogenesis of the disease by promoting the expression of pro-inflammatory cytokines (122). Targeting miR-155 with ASOs to inhibit its activity has been proposed as a therapeutic strategy to reduce inflammation in RA. Similarly, lncRNAs that modulate the expression of inflammatory genes can be targeted using small molecules or RNA interference to restore normal gene expression patterns (123). The modulation of ncRNAs represents a promising approach to fine-tuning the epigenetic regulation of inflammation, offering a new layer of control over immune responses (124).

The integration of epigenetic therapies with personalized medicine approaches holds significant potential for improving the treatment of inflammatory diseases. Personalized medicine aims to tailor treatments to the specific genetic and epigenetic profiles of individual patients, increasing the efficacy and reducing the side effects of therapies (125). Epigenetic profiling can identify specific epigenetic alterations that contribute to disease in a particular patient, allowing for the selection of targeted therapies that address these specific changes (126). For example, patients with RA who exhibit specific patterns of DNA methylation or histone modifications may benefit from targeted DNMTis or HDACis that reverse these epigenetic marks (127). The combination of epigenetic therapies with personalized medicine approaches offers the potential for more effective and individualized treatment strategies, particularly for complex diseases like RA and SLE (128).

Future directions in epigenetic therapy research include the development of more specific and potent epigenetic modifiers, the exploration of combination therapies, and the investigation of novel epigenetic targets (129). The discovery of new epigenetic enzymes and regulators will expand the repertoire of potential therapeutic targets, while combination therapies that include epigenetic drugs alongside traditional anti-inflammatory or immunosuppressive agents may enhance treatment efficacy (130). Additionally, the identification of novel epigenetic marks, such as RNA modifications and chromatin architecture alterations, could provide new avenues for therapeutic intervention (131). Continued research into the mechanisms of epigenetic regulation and its role in inflammation will be crucial for the development of next-generation epigenetic therapies (132).

## 10 Challenges and future directions in epigenetic research

**Figure 3:**
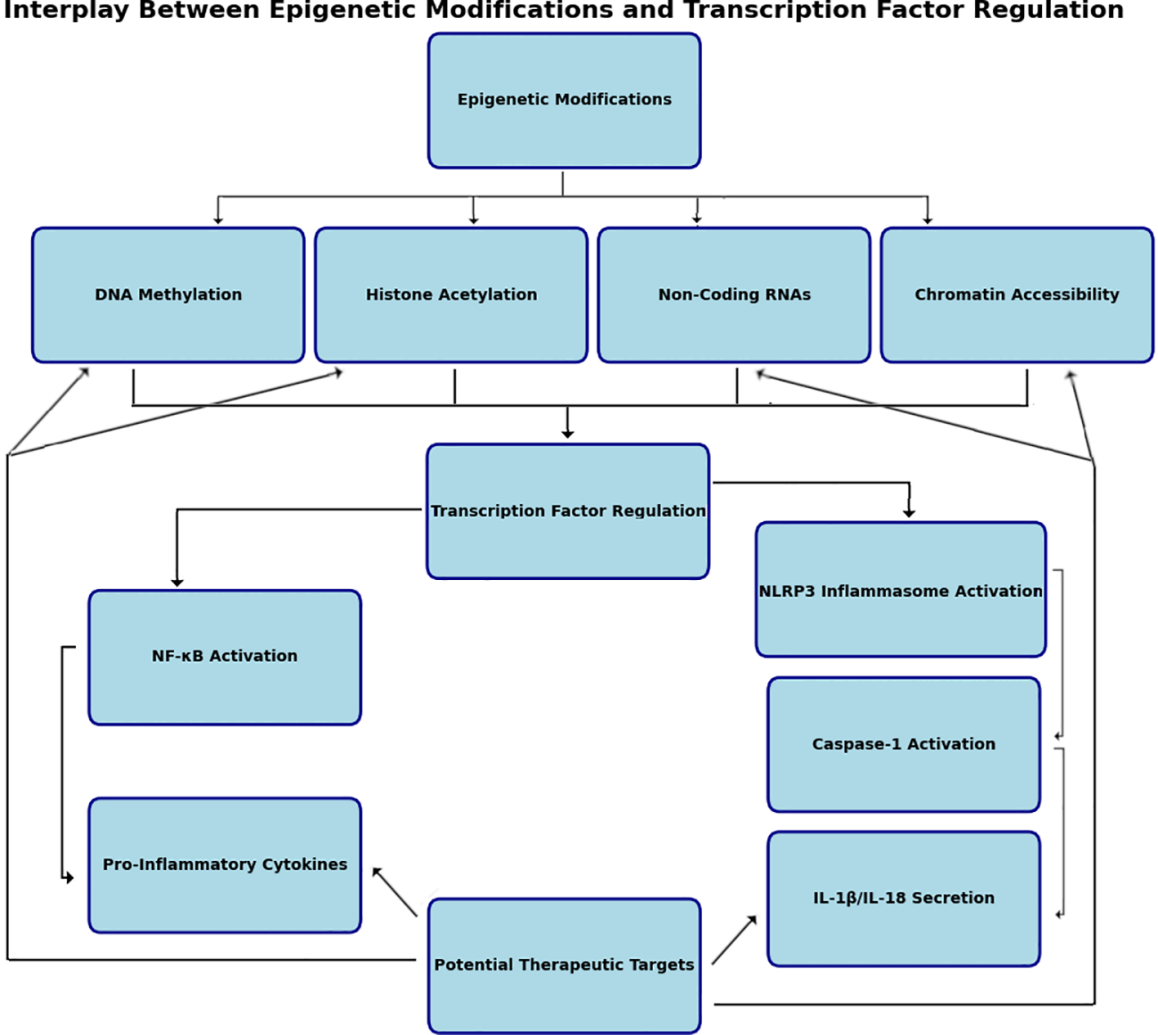
Interplay between epigenetic modifications and transcription factor regulation. Epigenetic regulation of transcription factors involves intricate interactions between histone modifications, DNA methylation, chromatin remodeling, and noncoding RNA mechanisms. Histone acetylation by histone acetyltransferases (HATs) and histone deacetylases (HDACs) alter chromatin structure therefore affecting transcription factor gene expression. DNA methylation mediated by DNA methyltransferases (DNMTs) adds methyl groups to CpG islands, suppressing transcription by preventing transcription factor binding to promoter regions. Noncoding RNAs, including microRNAs (miRNAs) and long noncoding RNAs (lncRNAs), regulate transcription factor activity by targeting mRNAs for degradation or altering chromatin states. miRNAs often function as negative regulators, while lncRNAs can act as scaffolds for chromatin-modifying complexes. These molecular processes converge to fine-tune transcriptional regulation, responding dynamically to cellular and environmental cues. Created in BioRender.com.

While significant progress has been made in understanding epigenetic mechanisms, translating these findings into clinical applications remains challenging. The complexity of epigenetic regulation, involving dynamic and context-specific interactions, complicates target identification (**Figure 3**). Additionally, the long-term effects and potential off-target consequences of epigenetic therapies are poorly understood, raising safety concerns. Gaps also exist in bridging preclinical findings to clinical outcomes due to interspecies differences and the lack of robust biomarkers for monitoring treatment responses (133). Future research should prioritize improving preclinical models, evaluating the durability of epigenetic modifications, and developing biomarkers to enhance the precision and safety of epigenetic therapies.

## 11 Conclusion

​In conclusion, the intricate relationship between epigenetics and inflammation underscores the critical role of epigenetic regulation in immune responses.​ The NLRP3 inflammasome and NF-κB signaling pathways are significantly influenced by various epigenetic mechanisms, including DNA methylation, histone modifications, and ncRNAs. Dysregulation of these processes is a hallmark of many chronic inflammatory and autoimmune diseases, offering insights into disease mechanisms and potential therapeutic targets. The development of epigenetic therapies represents a promising approach to treating inflammatory conditions, with future research in this field crucial for developing next-generation, personalized treatments. As our understanding of epigenetic regulation in inflammation deepens, it paves the way for more effective management of chronic inflammatory diseases.
